# Quantitative phase imaging unravels new insight into dynamics of mesenchymal and amoeboid cancer cell invasion

**DOI:** 10.1038/s41598-018-30408-7

**Published:** 2018-08-13

**Authors:** Ondřej Tolde, Aneta Gandalovičová, Aneta Křížová, Pavel Veselý, Radim Chmelík, Daniel Rosel, Jan Brábek

**Affiliations:** 1 Department of Cell Biology, Charles University, Viničná 7, Prague, Czech Republic; 2 Biotechnology and Biomedicine Centre of the Academy of Sciences and Charles University (BIOCEV), Průmyslová 595, 252 42 Vestec u Prahy, Czech Republic; 3 Central European Institute of Technology, Brno University of Technology, Purkyňova 656/123, 612 00 Brno, Czech Republic; 4 Institute of Physical Engineering, Faculty of Mechanical Engineering, Brno University of Technology, Technická 2896/2, Brno, 616 00 Czech Republic

## Abstract

Observation and analysis of cancer cell behaviour in 3D environment is essential for full understanding of the mechanisms of cancer cell invasion. However, label-free imaging of live cells in 3D conditions is optically more challenging than in 2D. Quantitative phase imaging provided by coherence controlled holographic microscopy produces images with enhanced information compared to ordinary light microscopy and, due to inherent coherence gate effect, enables observation of live cancer cells’ activity even in scattering milieu such as the 3D collagen matrix. Exploiting the dynamic phase differences method, we for the first time describe dynamics of differences in cell mass distribution in 3D migrating mesenchymal and amoeboid cancer cells, and also demonstrate that certain features are shared by both invasion modes. We found that amoeboid fibrosarcoma cells’ membrane blebbing is enhanced upon constriction and is also occasionally present in mesenchymally invading cells around constricted nuclei. Further, we demonstrate that both leading protrusions and leading pseudopods of invading fibrosarcoma cells are defined by higher cell mass density. In addition, we directly document bundling of collagen fibres by protrusions of mesenchymal fibrosarcoma cells. Thus, such a non-invasive microscopy offers a novel insight into cellular events during 3D invasion.

## Introduction

Cancer cell invasion is the crucial step in the process of metastasis formation, which is responsible for 90% of deaths in patients with solid tumours^1^. It is the only hallmark, which distinguishes benign and malign tumours^2^. To invade through the surrounding extracellular environment, cancer cells can utilize collective or individual migration. Collective invasion occurs when cancer cells maintain cell-cell contacts and stay in proximity with leading cells that proteolytically degrade the surrounding matrix, making way for the cell cohort to move forward^3^. Alike for collective migration, individually invading cells utilizing the mesenchymal invasion mode are characterized by their dependence on pericellular proteolysis, which enables cells to form tunnels in the extracellular matrix (ECM) for movement^4^. They are typically elongated with many actin-rich protrusions and cell-ECM adhesions. Conversely, amoeboid invasion does not rely on cell-ECM adhesions or proteolytical degradation of the ECM, instead amoeboid cells generate force by enhanced actomyosin contractility^5^ enabling them to squeeze through the pre-existing holes in the ECM. They are typically more rounded and exhibit membrane blebbing due to high hydrostatic pressure^6,7^.

Importantly, all modes of cancer cell invasiveness are interconvertible and could be employed by cancer cells in combination^8–10^. The ability of cancer cells to switch between modes of invasiveness is called plasticity and is an important issue related to the development of anti-invasive and anti-metastatic therapies^11–14^.

For the analysis of cancer cell malignant potential, manifested by invasiveness and plasticity, as well as for the analysis of the ability of various inhibitors to interfere with these processes, it is critical to observe the behaviour of cancer cells in a 3D environment. The most frequently used biologically derived 3D matrices for *in vitro* analysis of cancer cell invasiveness are Matrigel and 3D collagen gels^15^. Beside these gel-based matrices, more complex life-like matrixes derived from tissues are also used^16–18^.

However, there are general problems with lower optical transparency and light scattering in all these environments. These can be overcome by quantitative phase imaging (QPI) provided by coherence - controlled holographic microscopy (CCHM) due to inherent coherence gate effect (CGE), which makes possible imaging through a flowing turbid as well as static scattering medium. CGE is enabled by the spatially incoherent light^19^ used in CCHM. CGE differentiates among ballistic and strongly scattered light to eliminate the strongly scattered photons from contributing to the final image as noise^20–22^ (see Suppl. Fig. S1 and Supplementary text for details). Notably, the technique is non-invasive – no dyes or labels are used – and there is no halo artefact present, which typically disturbs imaging in Zernike phase contrast microscopy^23^. In addition, acquired images by CCHM are quantitative, making it possible to calculate cell dry mass in pg/μm^2^ from detected phase shifts^24–26^.

Here, we take advantage of CCHM to visualize in detail dynamics of cancer cell invasive behaviour, with focus on the interaction of cancer cells with collagen fibres, and utilize the quantitative information contained in the acquired images for analysis of cellular mass distribution and translocation.

## Results

### Establishment of a cell model for the study of amoeboid and mesenchymal migration

To compare features of amoeboid and mesenchymal invasion, we took advantage of the possibility to induce the mesenchymal-amoeboid transition by activation of the RhoA-ROCK pathway^27–29^. We used HT1080 fibrosarcoma cells stably expressing doxycycline-inducible constitutively active RhoA (caRhoA). Upon expression of caRhoA, the primarily mesenchymal cells gain a rounded phenotype with numerous membrane blebs. Their migration in 3D collagen is unaffected in the presence of GM6001, a broad-spectrum matrix metalloproteinase inhibitor, unlike migration of control mesenchymal cells that is stalled in such conditions (Suppl. Fig. S2), which corresponds to earlier descriptions of amoeboid and mesenchymal migration, respectively^4,30^. Using this cell model, we employed CCHM microscopy to describe in detail features of amoeboid and mesenchymal invasion.

### Mesenchymal migrating cells interact with surrounding ECM: examples of ruffling, bundling, and blebbing

**Figure 1:**
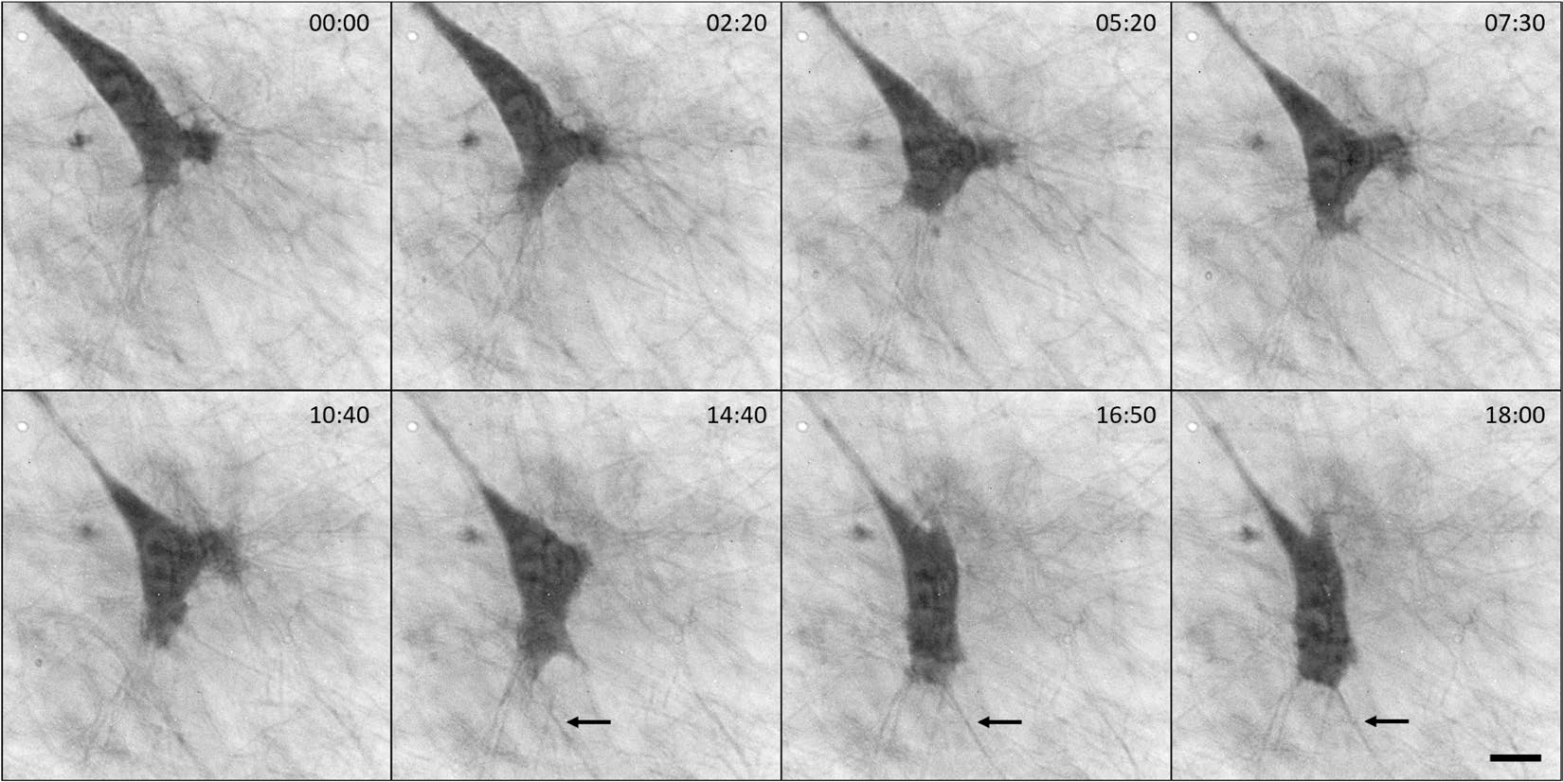
Migration of a mesenchymal cell within collagen matrix. Cells were embedded in 3D bovine collagen (1 mg/ml) and migration was observed by CCHM. Representative images with indicated times are shown. It can be noticed that the cell follows a thicker fibre (indicated by an arrow) once it gets to contact with it. The thickness of fibres was measured using ImageJ Plot Profile tool. The thick fibre has an average value 0.66 µm. The average value of the thinner fibres that are surrounding the cell in any direction or are in contact with the cell, is 0.39 µm (median 0.37 µm; n = 30). For full sequence see Suppl. Video V1. Scale bar: 10 μm.

**Figure 2:**
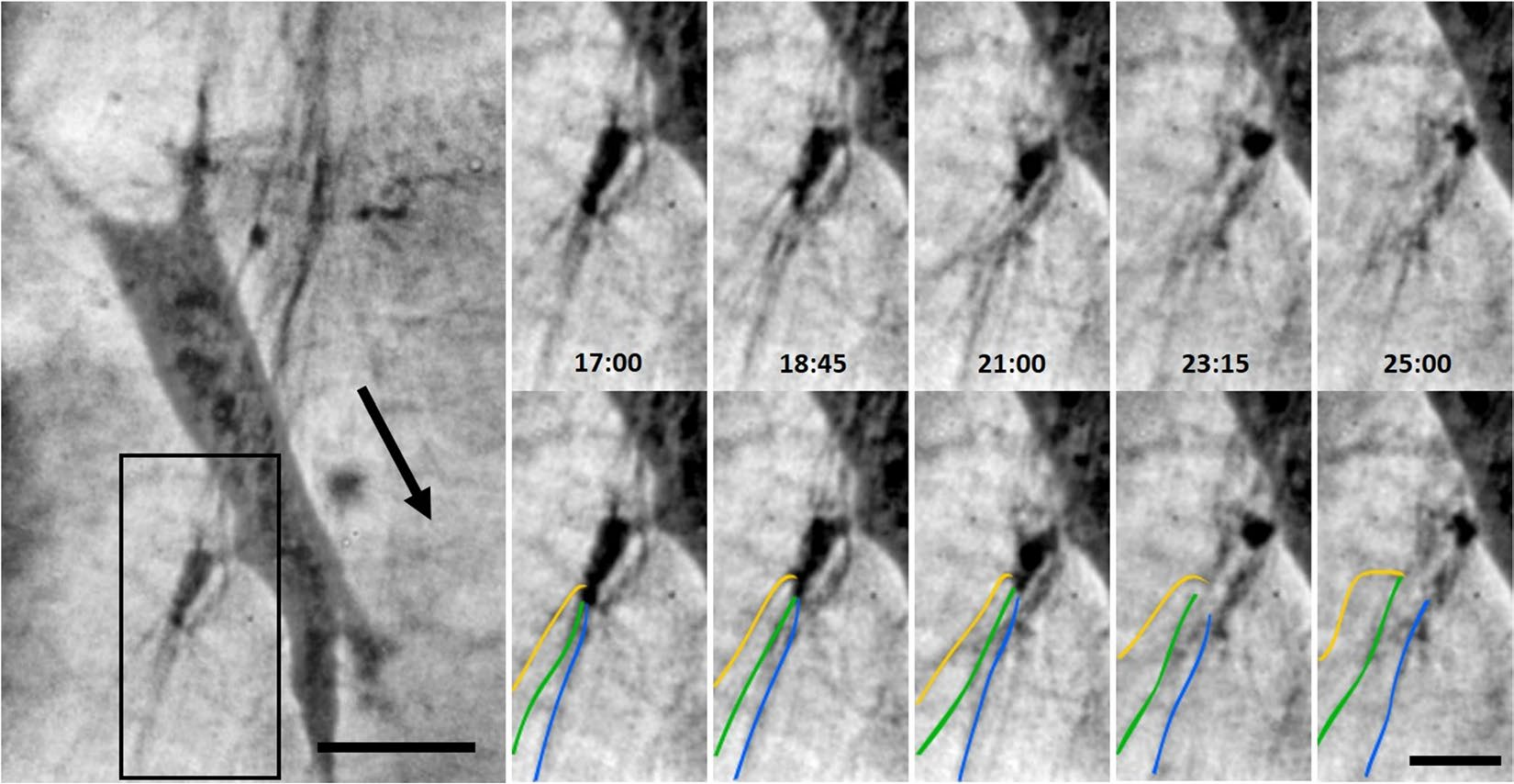
Bundling of collagen fibres by a mesenchymal cell. Cells were embedded within 3D bovine collagen (1 mg/ml) and images of migrating cells were acquired by CCHM. For better visualization of the fibre bundling, a situation when the cell retracts its pseudopodium, thus relaxing the fibres, is demonstrated. Left: Whole cell image, arrow indicates the direction of cell’s movement. Scale bar: 10 μm Right: A sequence of representative images with indicated time intervals corresponding to the Suppl. Video V4. Upper and bottom row show the same images, in the bottom row the three separate fibres are marked by different colours. Several fibres are apparently clustered by the cell protrusion, and a prominent displacement of these fibres is well seen after the protrusion is retracted and the fibres return to their relaxed positions. Scale bar 5 μm. Contrast was adjusted to higher degree in the inset.

Mesenchymal cell migration through tissue barriers requires pericellular remodelling of the ECM executed by cell-surface proteases, particularly membrane-type-1 matrix metalloproteinase (MT1-MMP). Invasive HT1080 fibrosarcoma cells were previously shown to coordinate mechanotransduction and fibrillar collagen remodelling by segregating the anterior force-generating leading edge containing β1-integrin, MT1-MMP and F-actin from a posterior proteolytic zone executing fibre breakdown. During forward movement, sterically impeding fibres are selectively realigned^31,32^. To study the interaction of mesenchymal cells with collagen fibres, we employed CCHM for imaging of HT1080 cells embedded within a fibrillar collagen matrix. We observed that during migration a mesenchymal cell followed a thick collagen fibre and evidently pulled on it (see Fig. 1 and Supplementary Videos V1–V4). When the mesenchymal cells were imbedded in collagen of lower density (0.5 mg/ml), the pulling on collagen fibres was more prominent and had larger impact on the collagen architecture (Suppl. Video V3). In addition, we observed that cells’ pseudopodia-like protrusions hold together several thinner fibres (Fig. 2, Suppl. Videos V4 and V5). We reckon this bundling of fibres is an active process serving to provide a more stable connection to the surrounding matrix, however further experiments would be needed for confirmation. CCHM also enabled observation of dynamic membrane ruffles at the cell front (Suppl. Fig. S3).

**Figure 3:**
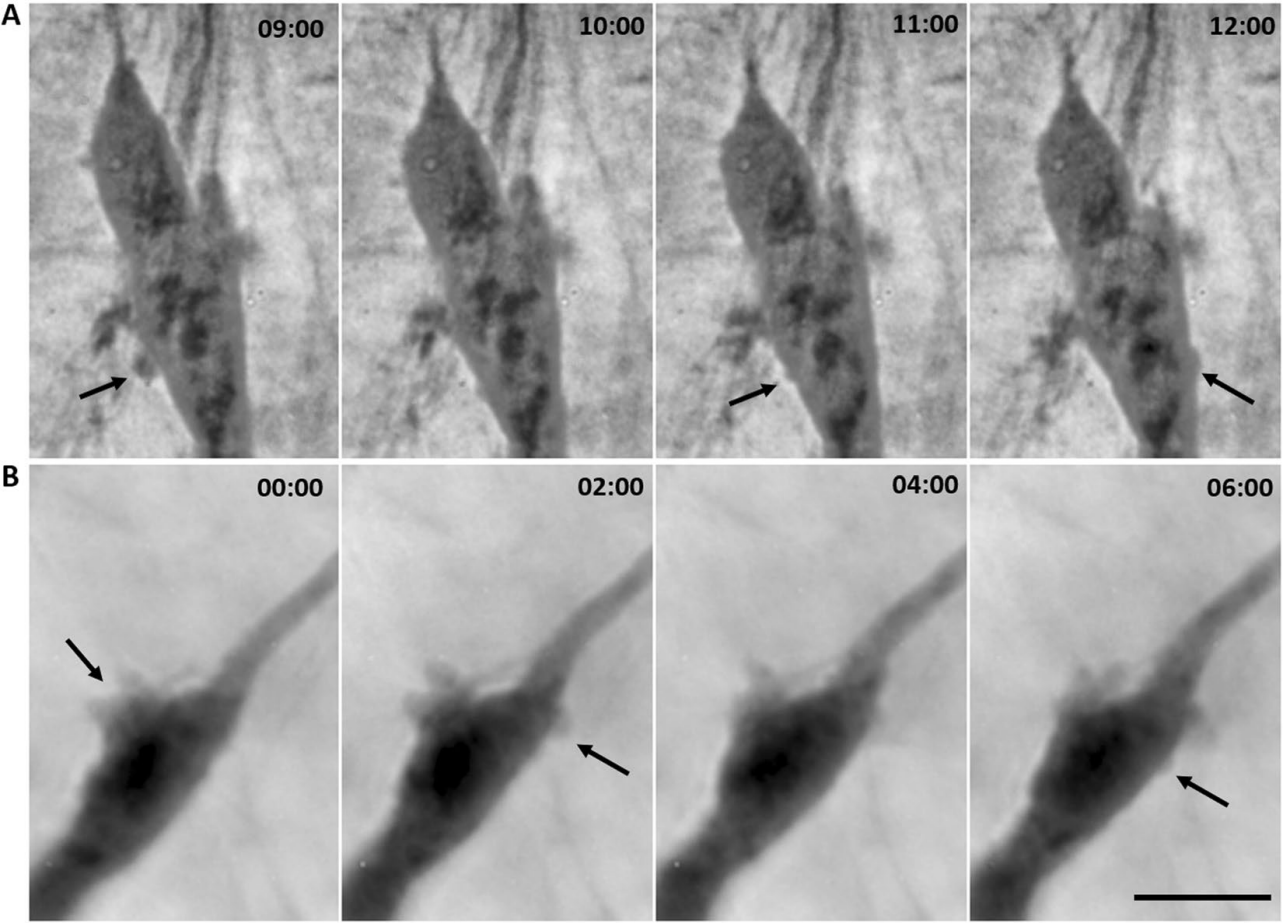
Perinuclear blebbing during mesenchymal invasion. Mesenchymal cells embedded in 3D bovine collagen (1 mg/ml) were imaged using CCHM. Arrows point towards membrane blebs formed around the nucleus. (**A**) Image sequence taken from Suppl. Video V6, which shows the dynamics of blebbing. For full sequence see Suppl. Video V4. (**B**) A second example of perinuclear blebbing during mesenchymal invasion. Scale bar: 10 μm.

Furthermore, we occasionally observed dynamic membrane blebbing of cells utilizing the mesenchymal invasion mode. The blebbing was temporary and observed around nuclei of cells whose movement was restrained by the surrounding matrix (Fig. 3 and Suppl. Video V6), making an impression that this blebbing serves to push away surrounding matrix and enable nuclear translocation.

### Cell mass is polarized and defines leading protrusions in mesenchymal cells

**Figure 4:**
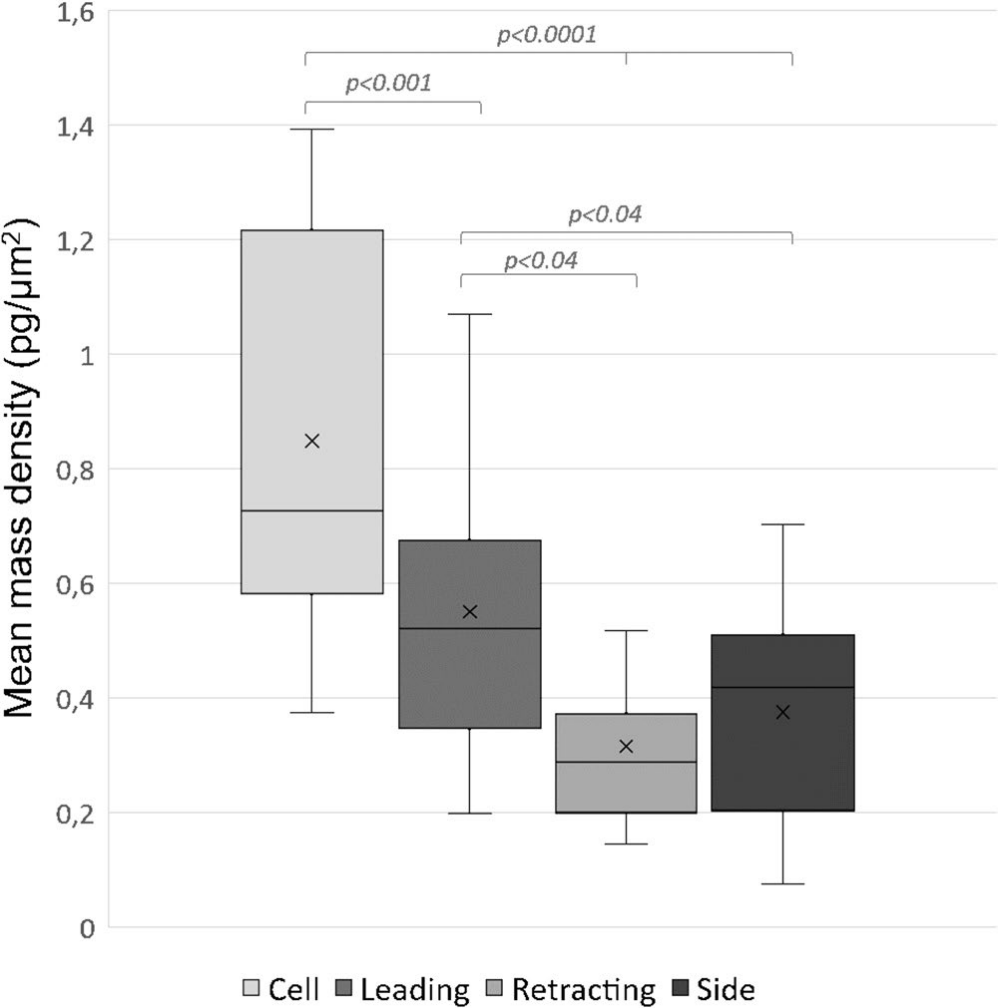
Analysis of mean mass density in protrusions of migrating mesenchymal cells. Images of mesenchymal cells embedded in 3D bovine collagen (1 mg/ml) were analysed to compute mean mass densities in whole cells and individual protrusions. Quantification results are expressed as box and whiskers (minimum to maximum) of 10 different cells. Cells were measured two to five times with time interval between measurements long enough for cells to substantially changed their morphology. Cell body (n = 22), leading (n = 20), retracting (n = 16), side (n = 50). Statistical significance was determined by one-way ANOVA followed by Tukey’s post-hoc test.

Next, we focused on cell mass distribution during mesenchymal migration. We computed mean mass densities of mesenchymal cells and their protrusions. The mean mass density within the cell body was significantly higher (0.85 pg/μm^2^, p < 0.001) than in the protrusions, reflecting mostly the high mass density of the nucleus and overall higher thickness of the cell body. Nevertheless, the leading protrusions have significantly higher (0.55 pg/μm^2^, p < 0.04) mean mass density compared to side protrusions and retracting protrusions (0.38 pg/μm^2^ and 0.32 pg/μm^2^, respectively) (Fig. 4). These results suggest that distribution of cell mass differs among individual protrusions to specify the leading protrusion, and in result defines the direction of invasion.

**Figure 5:**
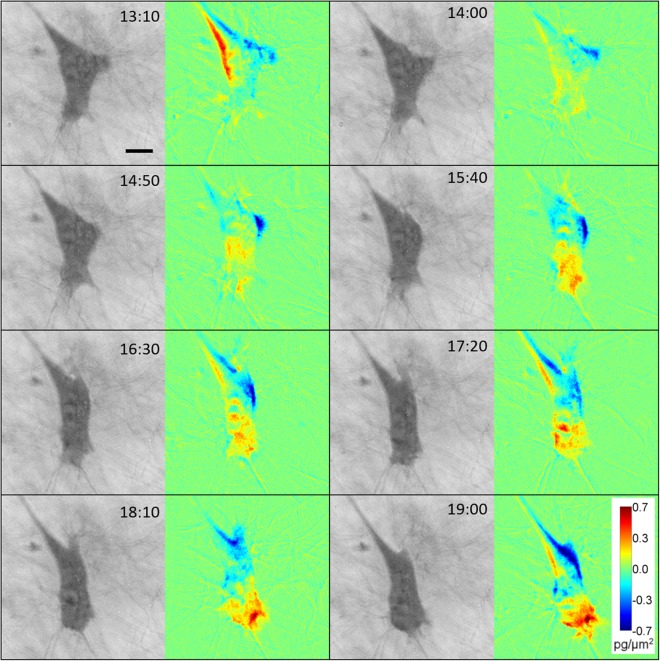
Analysis of cell mass during mesenchymal cell migration. Image sequence acquired by CCHM of a migrating mesenchymal cell. For full sequence see Suppl. Video V1. DPD show mass fluctuations between preceding and following images. The sequence demonstrates polarized cell mass distribution in a motile mesenchymal cell with a large influx of cell mass at cell front. Scale bar: 10 μm.

Moreover, postprocessing of images acquired by CCHM enables analysis of dynamic changes of cell mass distribution in migrating cells by calculating the dynamic phase differences (DPD) between consequent images (see Methods for more detail). The advantages of this method were demonstrated previously^33^. Analysis of cell mass differences during mesenchymal invasion highlighted the dynamics of membrane protrusions at the cell front and revealed a large influx of cell mass into the leading edge, clearly demonstrating the polarization of the cell, which corresponds to directionality of cell migration (Fig. 5).

### Various amoeboid cells phenotypes can be distinguished by cell mass distribution

To study amoeboid cancer cell behaviour in 3D collagen, we induced expression of constitutively active RhoA in HT1080 cells, which resulted in the gain of the amoeboid phenotype. It is well known that various amoeboid phenotypes can be employed, depending on the level of actomyosin contractility and adhesion^34,35^. By visualizing 3D migration of HT1080 cells with caRhoA using CCHM, we noticed that blebbing intensity is not constant and can be transiently replaced by formation of pseudopodia, denoting that various amoeboid states are acquired. We further describe these amoeboid phenotypes in more detail.

**Figure 6:**
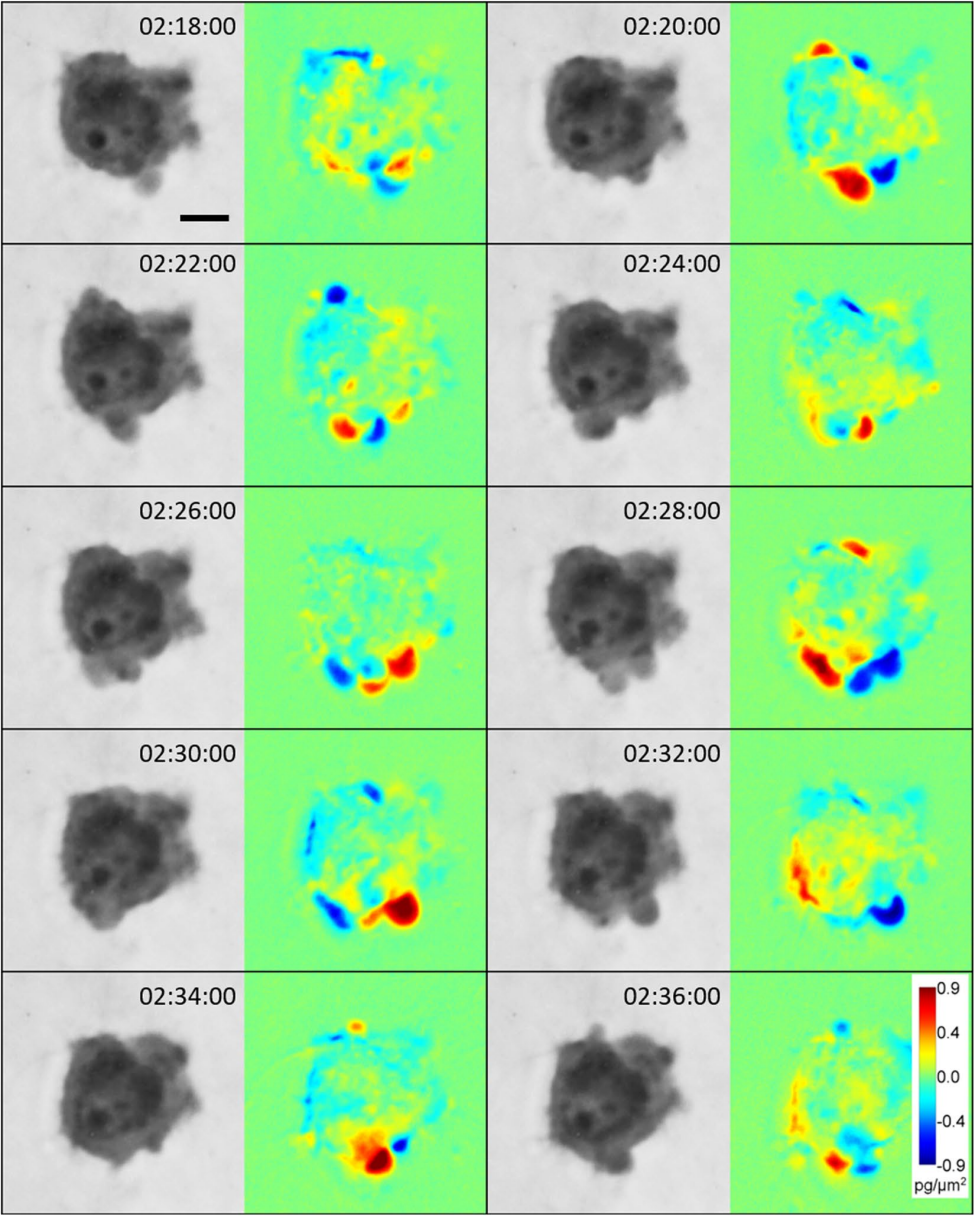
Dynamic cell blebbing observed in blebby-amoeboid cells. Image sequence acquired by CCHM of an amoeboid cell constricted in 3D rat-tail collagen (1 mg/ml), which led to enhanced blebbing. DPD show mass fluctuations between preceding and following images. It is clearly visible that the cell mass is unpolarized. For full sequence, see Suppl. Video V7. Scale bar: 10 μm.

We observed enhanced dynamic blebbing in amoeboid cells partly limited in movement, presumably due to ECM constriction. This phenotype resembles the blebby-amoeboid phenotype, during which numerous small membrane blebs form due to increased intracellular pressure. As observed by DPD, mass distribution inside the cell body during migration is less polarized compared to mesenchymal cells (Fig. 6 and Suppl. Video V7).

**Figure 7:**
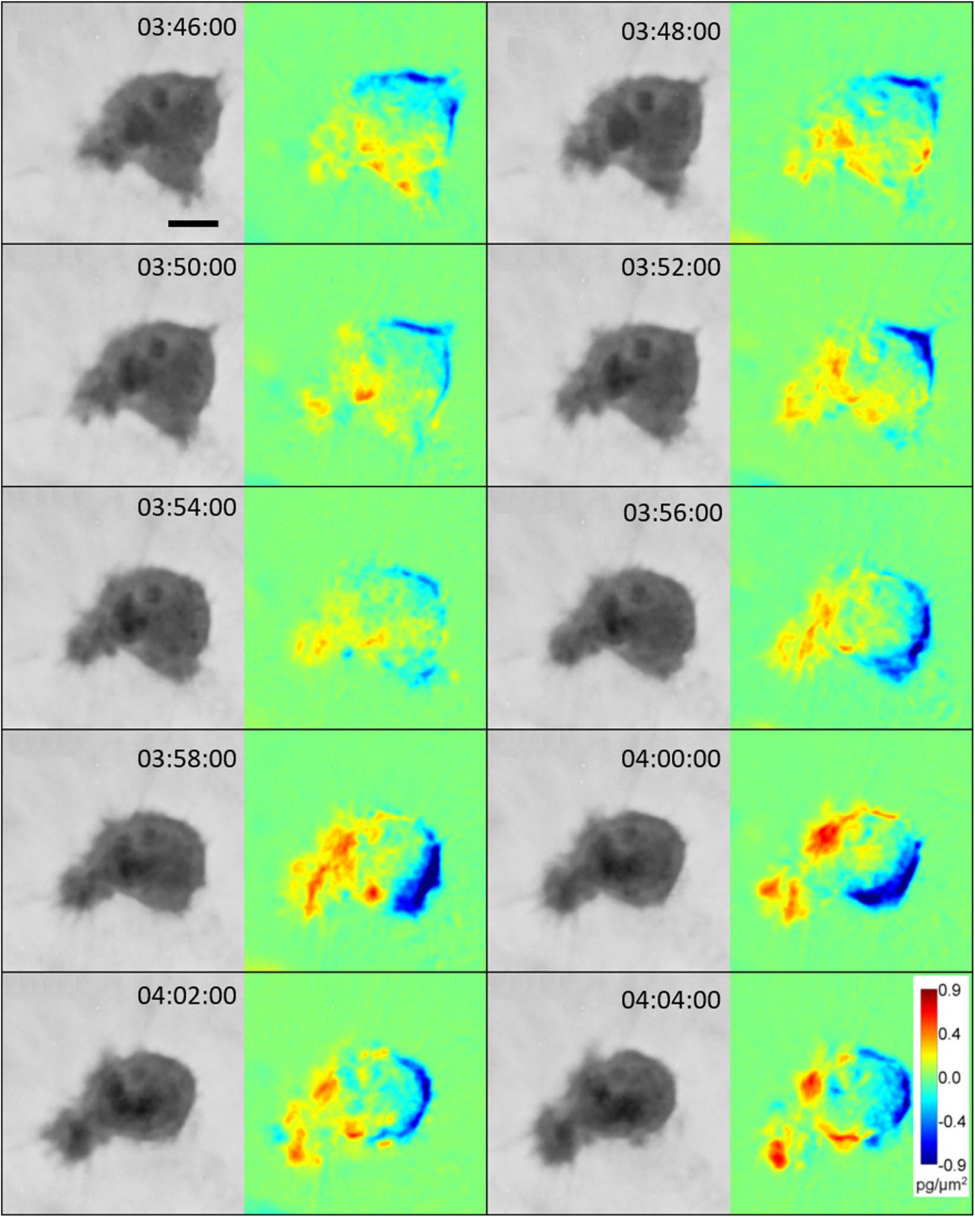
Analysis of cell mass during pseudopodal-amoeboid cell migration. Image sequence acquired by CCHM of a migrating amoeboid cell. For full sequence see Suppl. Video V7. DPD show mass fluctuations between preceding and following images. The sequence demonstrates polarized cell mass distribution in a pseudopodal-amoeboid cell, similar to cell mass polarization in mesenchymal cells (see Suppl. Fig. S3). Compare with unpolarized mass distribution during dynamic blebbing (Fig. 6). Scale bar: 10 μm.

**Figure 8:**
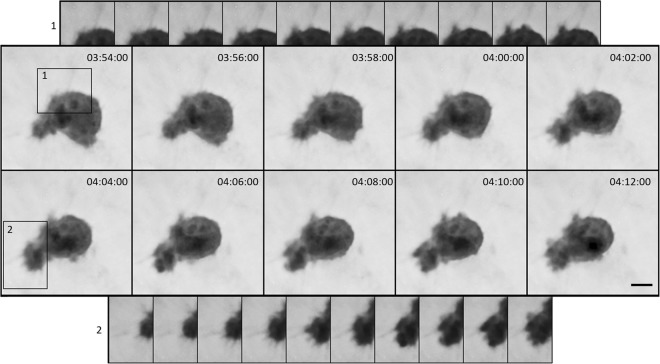
Interaction of an amoeboid cell with collagen fibres. Image sequence acquired by CCHM of a migrating amoeboid cell embedded in 3D rat-tail collagen (1 mg/ml). Transient contacts with surrounding fibres are visible in both inset 1 and 2. Notice that filopodia-like structures can form at the same site as blebs. Contrast was adjusted to higher degree in the insets. For full sequence, see Suppl. Video V7. Scale bar: 10 μm.

Blebbing intensity decreases when the cells switch to pseudopodal-amoeboid migration. Cells of this amoeboid phenotype are less rounded with several pseudopodia, resulting in a more motile state. Correspondingly, cell mass distribution is more polarized compared to blebby-amoeboid cells, with evident influx of cell mass into the leading front of the cell (Fig. 7). Moreover, as demonstrated for leading protrusions of mesenchymal cells, higher mean mass density also defines the leading pseudopodium during pseudopodal-amoeboid invasion. In fact, the mean mass density differentiates the leading pseudopodium from the lateral pseudopodium earlier than its size or weight individually (Suppl. Fig. S4). Furthermore, we directly observed that pseudopodal-amoeboid cells can promote transient contacts with surrounding fibres (Fig. 8).

**Figure 9:**
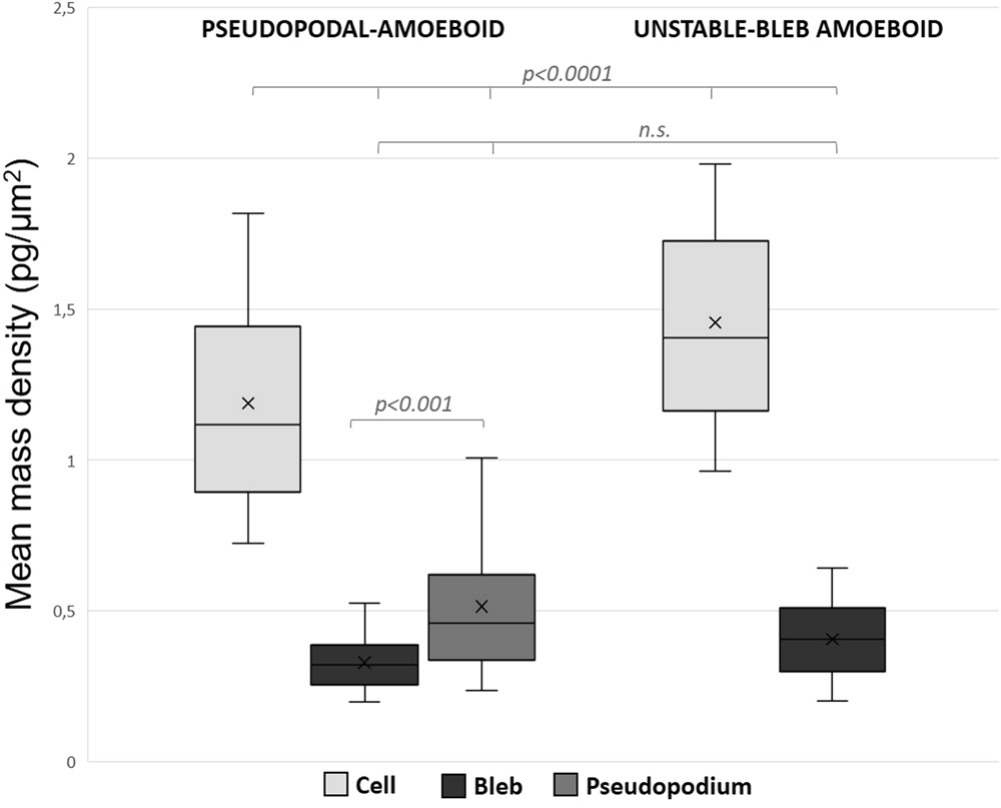
Amoeboid cell bleb measurements. Images of amoeboid cells embedded in 3D rat-tail collagen (1 mg/ml) acquired by CCHM were analysed to compute mean mass densities of cell bodies, pseudopodia and blebs in context of the observed amoeboid phenotypes. Overall, 10 cells and their pseudopodia and blebs were measured repeatedly with time interval between measurements long enough for cells to substantially changed their morphology. Cell body (n = 10), pseudopodium (n = 15), bleb (n = 88). Quantification results are expressed as box and whiskers (minimum to maximum). Statistical significance was determined by one-way ANOVA followed by Tukey’s post-hoc test.

To further characterize the two amoeboid phenotypes, we computed mean mass densities of individual cells, blebs and pseudopodia from acquired image sequences (Fig. 9). The mean mass density of blebby-amoeboid cells is significantly higher than of pseudopodal-amoeboid cells (1.45 pg/μm^2^ and 1.18 pg/μm^2^, respectively; p < 0.001). This corresponds to the more rounded cell body of blebby-amoeboid and more elongated body of pseudopodal-amoeboid cells. Interestingly, the mean mass density of amoeboid pseudopodia is 0.51 pg/μm^2^, which is very similar to pseudopodia of mesenchymal cells (0.55 pg/μm^2^). The mean mass densities of blebs were not significantly different in case of blebby-amoeboid cells and pseudopodal-amoeboid cells (0.41 pg/μm^2^ and 0.33 pg/μm^2^, respectively) and were 2.8x lower than mean mass densities in cell bodies in both cases. Overall, the variance of mean mass densities in blebs was much lower (coefficient of variation (C_v_) 30%) compared to variance of bleb area (C_v_ 65%) or weight (C_v_ 92%).

**Figure 10:**
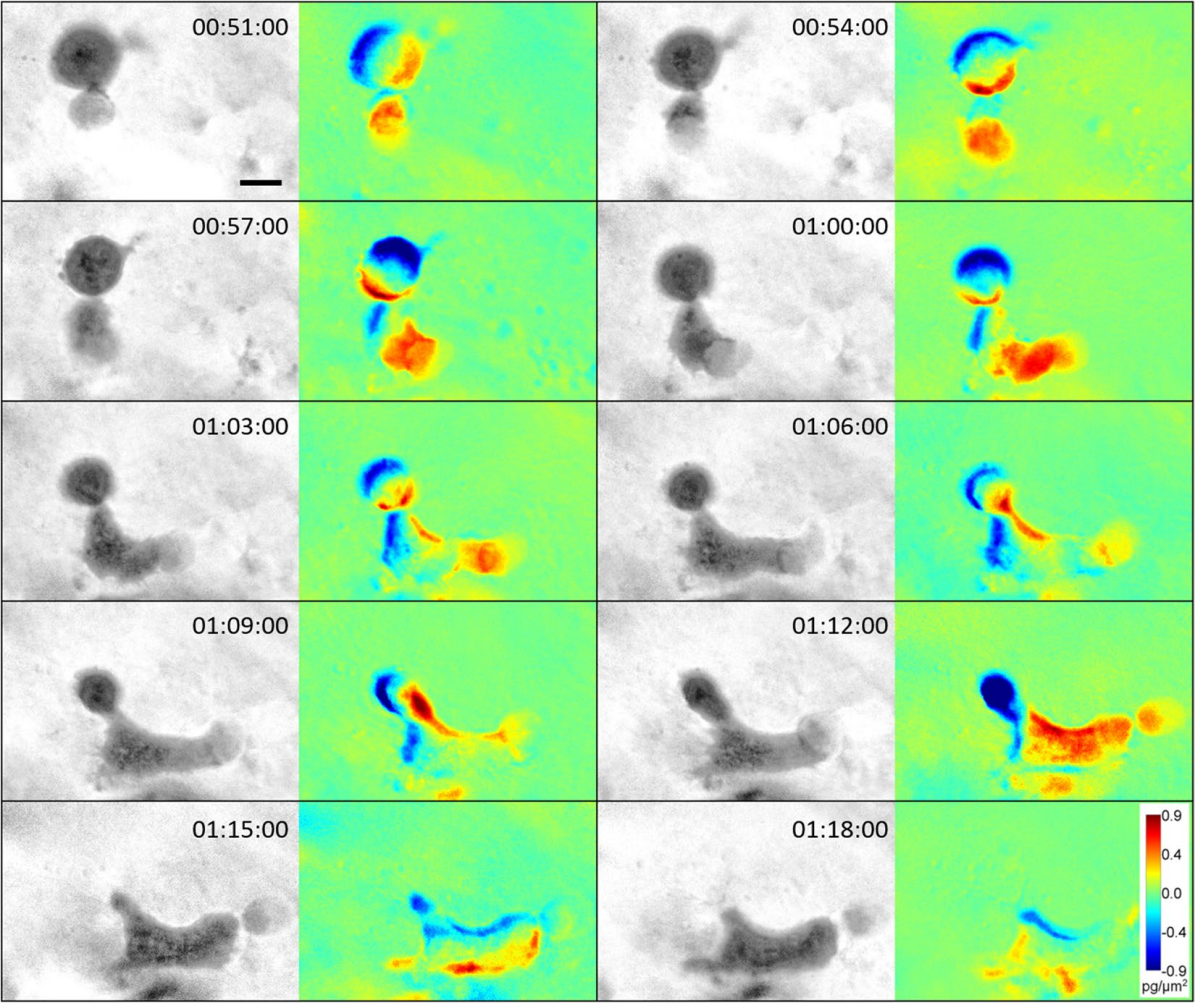
Analysis of cell mass of an amoeboid cell during translocation through a narrow pore. Image sequence acquired by CCHM of an amoeboid cell translocating through a narrow pore in 3D rat-tail collagen (1 mg/ml). To invade through the pore, the cell dynamically deforms its cell body and nucleus. DPD depict mass fluctuations between preceding and following images. As shown by DPD, the largest influx of cell mass occurs between time 01:12:00 and 01:15:00, corresponding to the time of nuclear translocation. For full sequence see Suppl. Video V8. Scale bar: 10 μm.

The plasticity of amoeboid cells is provided by high deformability of the cell body, which is limited by stiffness of the cell nucleus^36,37^. Therefore, we were interested in cell mass distribution within an amoeboid cell that undergoes large deformation of its body and nucleus while overcoming a constriction in the ECM (Suppl. Video V8). To pass through the narrow constriction, the cell initially formed a large membrane bleb protruding through the pore in the collagen matrix. The bleb later transformed into a stable pseudopodium, which increased in size until the remaining cell body was able to proceed. As demonstrated by DPD, the translocation of the nucleus is limiting for translocation of the whole cell, since the largest influx of cell mass occurs after the nucleus is able to pass (Fig. 10).

Taken together, our results demonstrate the ability of CCHM to not only visualize features of cell invasion in detail, but also directly analyse cell mass densities and distribution.

## Discussion and Conclusions

Noninvasive quantitative phase imaging through scattering media is an important task in biomedical research and draws the *in vitro* model applications nearer to real situations in living organisms. Detailed visualization of live cells in 3D collagen as reported here was enabled by coherence gate effect, which is characteristic of quantitative phase imaging in the spatially incoherent light. Taking advantage of the CCHM we investigated in detail the features of cancer cells utilizing either the amoeboid or mesenchymal invasion mode and analysed cell mass distribution changes within the migrating cancer cell.

First, we observed the dynamic interaction of cancer cells with surrounding fibres. We directly documented migration of a mesenchymal cell toward a thicker fibre in 3D collagen. The tendency of cells to follow thicker fibres was shown previously^17,38,39^, moreover it was speculated that thick collagen fibres serve as a migration highways for cancer cells within tissues^40^. An interesting observation visualized by CCHM was bundling of several collagen fibres together by pseudopodia-like protrusions of a mesenchymal cell (Fig. 2). We can speculate that the bundling serves to ensure a more stable connection to the surrounding matrix by providing focal/fibrillar adhesion sites or by alternative strategies, such as forming clathrin/adaptor protein 2 lattices^41^.

Further, we observed dynamic filopodia at the leading edge of mesenchymal cells (Suppl. Fig. S3). Filopodia can act alone or in combination with blebs, lobopodia or lamellipodia in 2D and 3D environments^42^. We assume that these dynamic filopodia are components of membrane ruffles - structures initially described in detail in 2D, but proven to form in 3D environments as well using other microscopy techniques^42–44^. Peripheral ruffles assemble at the leading edge of motile cells, where filopodia act to sense the local microenvironment^45^. It was shown that activated β1 integrins are localized to the tips of filopodia along the leading edge^46^, as well as other components of focal adhesions^47^. Actin-based movement of primed integrins along the leading edge suggests a “sticky fingers” mechanism to probe for new adhesion sites and direct migration^46^. Thus, we assume that formation of 3D ruffles, as visualized by CCHM, share a similar function as their 2D equivalents.

Notably, cell-ECM contact was observed also in case of amoeboid migration (Fig. 8), which is often considered to be independent of ECM adhesions^48–50^. Adhesions of amoeboid cells differ from those of mesenchymal cells - they are often depleted of integrins and instead formed by alternative adhesive molecules such as cell surface receptors^49^. Our observations of transient cell-ECM fibres contacts correspond with previous reports that show that amoeboid cells employ only short-lived contacts with collagen fibres that do not result in fibre cleavage^4^ and enable rapidly adaptive migration^51^.

Imaging typical features of mesenchymal and amoeboid cells unveiled that these elements can be shared among all individually invading cancer cells. As mentioned above, we observed cell-ECM contact, typical of mesenchymal cells, also in amoeboid cells. Moreover, we repeatedly observed dynamic membrane blebbing of amoeboid cells and, surprisingly also mesenchymal cells, at sites where ECM constrictions impede further movement (Figs 3, 6, Suppl. Video V6 and V7). We hypothesize that the purpose of this enhanced blebbing is to push away surrounding material to unravel a path wide enough for the stiffest part of the cell to translocate- its nucleus. Because this phenomenon was observed also in mesenchymal cells that produce ECM-degrading enzymes, we assume that under certain conditions, temporally enhanced blebbing is preferred over ECM proteolysis. This phenomenon was already observed in migrating macrophages^52^, but to what extent it facilitates cancer cell invasion remains to be clarified.

The quantitative character of CCHM images allows to measure mean cell mass densities. We show the method is applicable for measurements of cells inside a 3D environment and can be used to determine mean mass densities of individual cell regions. Other methods utilized for cell mass measurements, such as cantilever mass sensors or microfluidic channels, only perform whole cell measurments^53–55^.

For analysis of cell mass translocation during cancer cell invasion, we applied DPD method that reveals influx or decrease of mass from subsequent images. In migrating mesenchymal cells, cell mass increases towards the leading edge and overall maintains this polarized distribution during invasion (Fig. 5). We also show that mean mass densities are higher in leading protrusions than side or retracting protrusions, which can serve to define the direction of invasion (Fig. 4).

On the other hand, amoeboid cells display dynamic changes in cell mass distribution. During blebby-amoeboid invasion, the cell mass translocation is largely unpolarized inside the cell (Fig. 6 and Suppl. Video V7). The gain of a pseudopodal-amoeboid phenotype results in more polarized cell mass, with mean mass density increasing in the leading protrusion (Fig. 7, Suppl. Fig. S4 and Suppl. Video V7). This indicates that increased cell mass redistribution to the leading protrusion is a characteristic common for both mesenchymal and amoeboid cells.

Notably, mean mass density in blebs does not change considerably with increasing bleb size and maintains a value approximately 2.8x lower than the mean mass density of the whole cell in case of both blebby- and pseudopodal- amoeboid migration (Fig. 9). We also demonstrate, that cell mass is unequally distributed during invasion of a cell through a narrow pore until the nucleus itself is able to pass (Fig. 10).

These results confirm the potential of CCHM to reveal novel characteristics of cancer cell invasion and contribute to our understanding of invasive mechanisms. Nevertheless, QPI techniques have already yielded several findings of cellular behaviour^56^. For example, dynamic phase differences were employed for the visualization of changes in distribution of cell dry mass upon nutritional stress^57^, an approach, which was later elaborated as an evaluation method^33^. QPI also permits to differentiate between cell death caused by apoptosis and oncosis^58^ or reveal necrosis^59^. Further, it enabled to observe entosis as a way of cancer cell survival under stress^60^. CCHM analysis of primary cells derived from head and neck squamous cell carcinoma biopsy proposed a dynamic phenotype of carcinoma cells to be a criterion for the recognition of cancer while still alive in primary culture^61^. Such recognition was achieved by exploitation of the possibility to simultaneously measure cell migration and growth by evaluating gain of cell dry mass. A different study demonstrates that digital holographic microscopy can distinguish the metastatic potential of melanoma cells^62^.

Recently, a new category of agents focused on cancer metastasis prevention was proposed and named migrastatics^14^. The search for migrastatics will require to test the effect of candidate agents on the behaviour of cells in 3D environments. According to results obtained from both 2D cultures^61^ and here in 3D, we propose CCHM as a suitable approach for the early stages of potential migrastatic drugs assessment, mainly due to its ability to clearly visualize cell invasion without any need of labelling.

However, high-throughput analysis of cell invasive behaviour requires the establishment of an automated evaluation method^63^. Automatic classification of cells based on QPI has already shown to be possible in 2D environments^64^. The quest is now to enable similar evaluation in 3D. With the incorporation of DPD method and development of proper machine learning algorithms, it is considered feasible. Our results suggest that mesenchymal cells maintain polarized cell mass during migration, whereas amoeboid cells switch among the less-polarized (blebby-amoeboid) and more-polarized (pseudopodal-amoeboid) states. Moreover, amoeboid cells form numerous membrane blebs in both states, while mesenchymal cells form blebs only temporally. Thus, an algorithm able to include both QPI and DPD parameters could differentiate among the various invasion modes given images with sufficient time resolution are provided. In addition, DPD enables to easily determine the direction of cell movement by analysis of mass polarization and can monitor the migratory process, represented by cycles of high and low mass changes during rear retraction (Suppl. Fig. S5).

Altogether, we took advantage of the coherence gate effect produced by coherence - controlled holographic microscopy to study invasive behaviour of cancer cells in 3D collagen. Observations presented here using CCHM demonstrate that cancer cells can temporally and dynamically utilize characteristics of both the amoeboid and mesenchymal phenotype and adjust their modes of invasion according to current conditions. It is evident that CCHM is a valuable tool for studying the plasticity of cancer cell invasion.

## Methods

### Cells, culture and material

Human fibrosarcoma cells HT1080 and HT1080 with constitutively active RhoA (RhoA G14V) stable cell line were routinely cultured in standard conditions (37 °C, humidified atmosphere with 5% CO_2_) in full DMEM medium (Life Technologies) with 4.5 g/l L-glucose, L-glutamine, and pyruvate, supplemented with 10% fetal bovine serum (Sigma Aldrich) and 0.1% ciprofloxacin (Sigma Aldrich). The HT1080 RhoA G14V stable cell line was prepared and provided by Vladimír Čermák, PhD.

### Microscopy

Quantitative phase imaging (QPI) was performed using Q-PHASE (TESCAN Brno, s.r.o.) a multimodal holographic microscope based on CCHM technology. A halogen lamp and interference filter with a central wavelength 650 nm and 10 nm FWHM were employed as a light source. For observations Nikon Plan 10×/0.3, Nikon Plan Fluor 20×/0.5 and Nikon Plan Apo 40×/0.95 objectives were used. The hologram was taken by a single shot with a CCD camera (XIMEA MR4021MC). Quantitative phase image was computed in real time by implemented Q-PHASE control software. For more information see Supplementary text.

### 3D collagen matrix

Cells were trypsinized, centrifuged and resuspended at concentration 1 × 10^6^ cells/ml in phenol red free DMEM supplemented with 10% FBS. Buffered solution (composed of 1x DMEM, 0.375% NaHCO_3_, 8.5 mM NaOH, 15 mM Hepes, 0.1% ciprofloxacin and 1 mM folic acid final concentration) was mixed with ddH_2_O and collagen (Cultrex; rat tail or Biochrom; bovine; both 4 mg/ml) depending on final collagen concentration. Cell suspension was added in ratio 1:10. For the analysis of mesenchymal invasion we used 1 mg/ml bovine collagen solution (unless stated otherwise), which led to clearly visible thicker fibres. Rat-tail collagen 1 mg/ml was employed for the analysis of amoeboid invasion.

The mixture was placed either in a µ-Slide Angiogenesis well (Ibidi) or a custom-made chamber created from Mattek dishes. Gel solidification was performed at 37 °C for at least 1 hour, after which the Ibidi µ-Slide or custom-made chamber was filled entirely with medium (phenol red free DMEM supplemented with 10% FBS, 10 mM BES buffer, and 7.5 mM Hepes buffer) and covered with a cover slip (22 × 40 mm), or chamber lid, respectively, to avoid the formation of a meniscus that would disrupt the interference and quality of the phase image. Prior to observation, the chambers were transferred to the Q-PHASE microscope placed in a box tempered to 37 °C.

### Image processing

Images and live cell videos were obtained using Q-PHASE control software (TESCAN Brno, s.r.o.). These quantitative phase images contain information about cell dry mass density in each pixel. The total mass of the cell is given as a sum of pixel values in the area of the cell, the mean cell mass density is calculated as a sum of individual pixel values in the cell divided by the area of the cell. For the analysis of mean mass density values of different regions, acquired images were analysed in ImageJ. Each region of interest (cell, bleb, protrusion) was marked manually and measured. Mean values in pg/μm^2^ were plotted into graphs.

For step by step analysis of cell mass changes, the difference in mass distribution between two images was calculated by dynamic phase differences (DPD)^33^. Briefly, DPD visualizes the difference between selected subsequent images, which is calculated by subtracting the previous from the following image. The resulting plus difference indicates proportionally the mass gain in red colour, while the minus difference depicts mass loss in blue colour. Small differences are indicated as noise around zero value, which is illustrated in light green colour. Because the changes of cell mass are distinctly higher than those of the surrounding collagen, they appear in red or blue colours, whereas the collagen milieu is represented in light green colour.

For presentation purposes only, the contrast of quantitative phase images was enhanced by non-linear filtration in ACC (Adaptive Contrast Control © software by SOFO).

Post processing for visualisation of membrane ruffles was performed by ImageJ. Pseudo-coloured images were prepared using a macro. In brief, pixels with grey value above a manually set threshold were copied to a new channel and coloured using LUT. Subsequently, a threshold was used for marking the pixels on the cell´s periphery and obtained masks were saved as binary images. The assembly and colouring of layers were performed in Photoshop.

### Statistical analysis

Statistical analysis of significance was done in Prism 6 software (GraphPad Software, Inc.). For analysis of mean mass densities one-way ANOVA followed by Tukey’s post-hoc test was used. Coefficients of variation were calculated standardly as ratio of standard deviation to mean value and are expressed as percentage.

## Electronic supplementary material

Supplementary info

Video 1

Video 2

Video 3

Video 4

Video 5

Video 6

Video 7

Video 8
