# Supplementary material for: Quantitative phase imaging unravels new insight into dynamics of mesenchymal and amoeboid cancer cell invasion
Source: Sci Rep. 2018 Aug 13;8:12020. doi: 10.1038/s41598-018-30408-7 (PMC6089916; doi:10.1038/s41598-018-30408-7)
Supplement: Supplementary file 1 — Supplementary info [file 41598_2018_30408_MOESM1_ESM.pdf]

SUPPLEMENTARY INFORMATION

Quantitative phase imaging unravels new insight into dynamics of mesenchymal and amoeboid cancer cell invasion

Ondřej Tolde [1,2#], Aneta Gandalovičová [1,2#], Aneta Křížová [3,4], Pavel Veselý [3], Radim Chmelík [3,4], Daniel Rosel [1,2] and Jan Brábek [1,2]

[1]Department of Cell Biology, Charles University, Viničná 7, Prague, Czech Republic

[2]Biotechnology and Biomedicine Centre of the Academy of Sciences and Charles University (BIOCEV), Průmyslová 595, 252 42, Vestec u Prahy, Czech Republic

[3]Central European Institute of Technology, Brno University of Technology, Purkyňova 656/123, 612 00, Brno, Czech Republic

[4]Institute of Physical Engineering, Faculty of Mechanical Engineering, Brno University of Technology, Technická 2896/2, Brno 616 00, Czech Republic

# These authors contributed equally to the work.
*Correspondence: jan.brabek@natur.cuni.cz (Jan Brábek)

Supplementary text:

CCHM setup

In layman's terms, coherence gate effect permits only the use of image information carrying photons, which are the ballistic (un-scattered) or snake-like (slightly scattered) photons for image formation by interference. Each of them originates from single emitting atom making the single illuminating ray until it is split (BS in Fig. S1) into the reference and object rays, the coherent twins (yellow and blue rays respectively, in Fig. S1). Thus, they can contribute to building the final image only by interfering with the twin partner provided they meet at detector level (D in Fig. S1) within the coherence volume. The coherence volume is very small in partially coherent systems compared to its large size in coherent (laser driven) systems in which every ray can interfere with any other ray. Strongly scattered rays (red rays in Fig. S1) cannot contribute to image formation as they miss the coherence volume and so their twin reference partners for interference get lost. In result, no disturbance of the final image by noise generated by strongly scattered rays, as it happens in coherent systems, occurs.

Supplementary Figures with legends

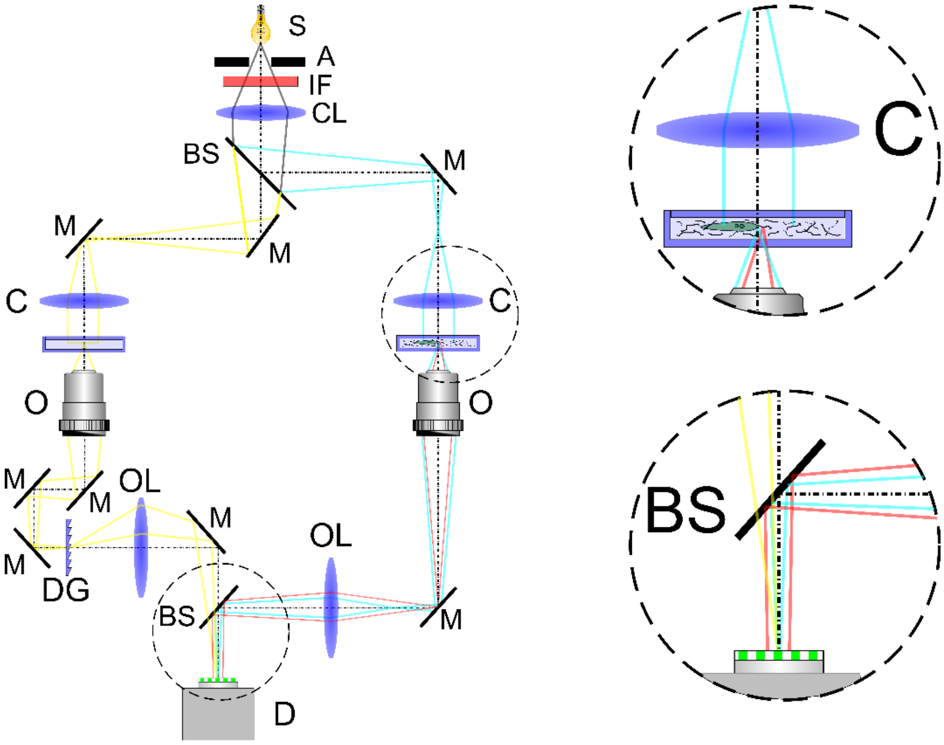

Figure S1. Detailed description of coherence-controlled holographic microscope quantitative phase imaging with coherence gate effect.

The light from source S passes through an aperture A and interference filter IF. The beam splitter BS divides it into two separated optical paths - the object arm (blue) and the reference arm (yellow) of the interferometer. Both arms consist of condenser C, objective O and output lens OL. The sample with scattering medium is placed in the object arm, only the reference object without scattering medium is in the reference arm. The diffraction grating DG is located in the reference arm. The reference beam and the object beam that include only ballistic (un-scattered) light recombine in the output plane and create an interference fringes pattern. The primary beam, reference beam and corresponding object beam are depicted in black, yellow and blue colour, respectively. Green interference fringes are produced by combination of yellow reference beam with blue ballistic object beam. Strongly scattered light (red colour in the figure) does not contribute to the interference because it has no corresponding (mutually coherent) reference beam to interfere with (see detail at the bottom right). Camera D captures the interference fringes pattern as holograms. The upper detail shows the formation of strongly scattered light at an ECM fibre.

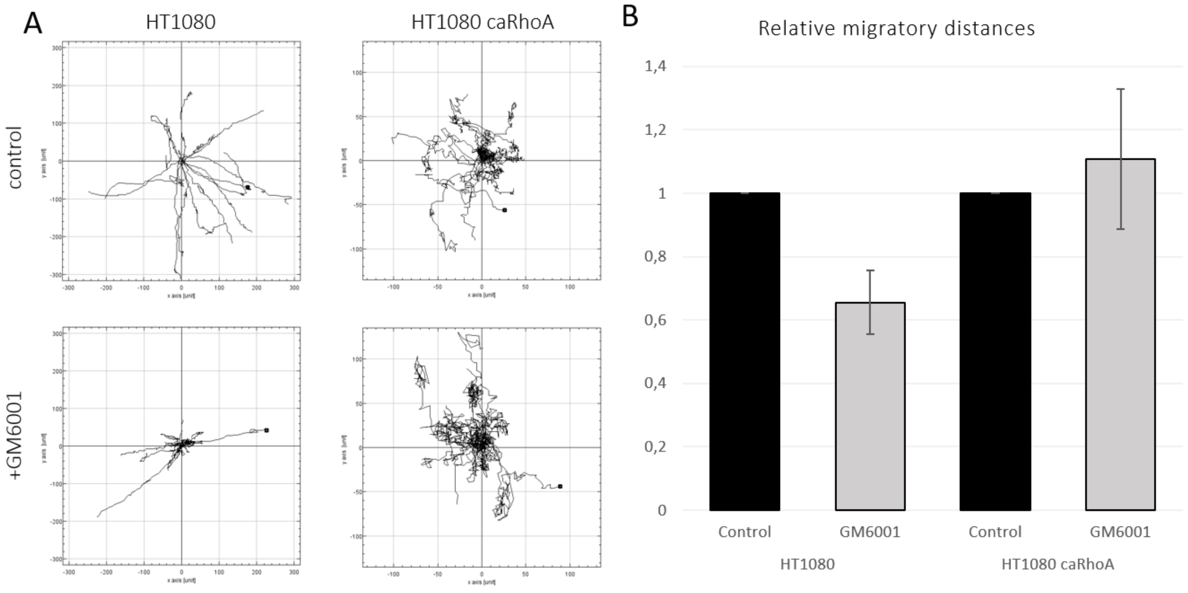

Figure S2. Migratory distances of HT1080 cells of the mesenchymal and amoeboid phenotype.

HT1080 cells and HT1080 cells expressing constitutively active RhoA were embedded in rat-tail collagen (1mg/ml) with or without GM6001. 12 hours later, migration of cells of the mesenchymal phenotype (HT1080) and amoeboid phenotype (HT1080 caRhoA) was monitored by wide-field microscopy for 15h. Three independent experiments were evaluated. A) Track plots were generated using Chemotaxis Tool in ImageJ. For each condition, one representative track plot is shown. B) Quantification of relative migratory distances.

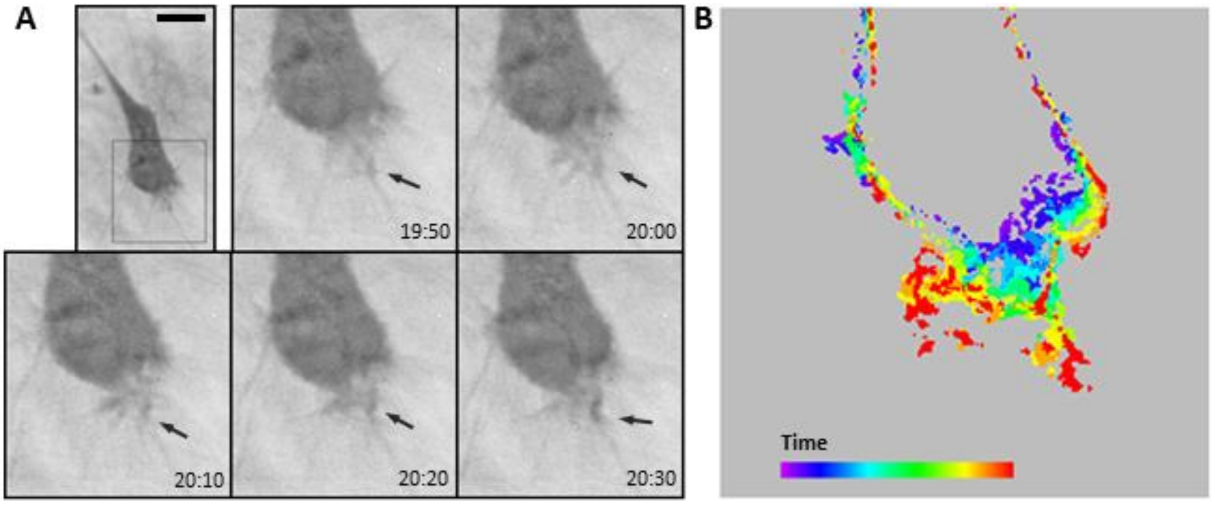

Figure S3. Membrane ruffles in 3D collagen. Image sequence acquired by CCHM of a migrating mesenchymal cell showing membrane ruffles at the cell´s front. A: Representative images are taken from Supplementary video V1 with time points corresponding to the video. Arrows points towards presumed membrane ruffles, an enrichment of filopodia at the cell‘s leading edge. B: The dynamics of membrane filopodia protrusions at the cell´s front visualized in colours. The rainbow colours represent cell periphery at different time points (14 in total) in chronological order, with violet representing the periphery from the earliest frame and red the latest. Pseudo-coloured representation highlights the most dynamic areas of cell periphery that correspond to the filopodia ruffles indicated in A. Scale bar: 10 µm

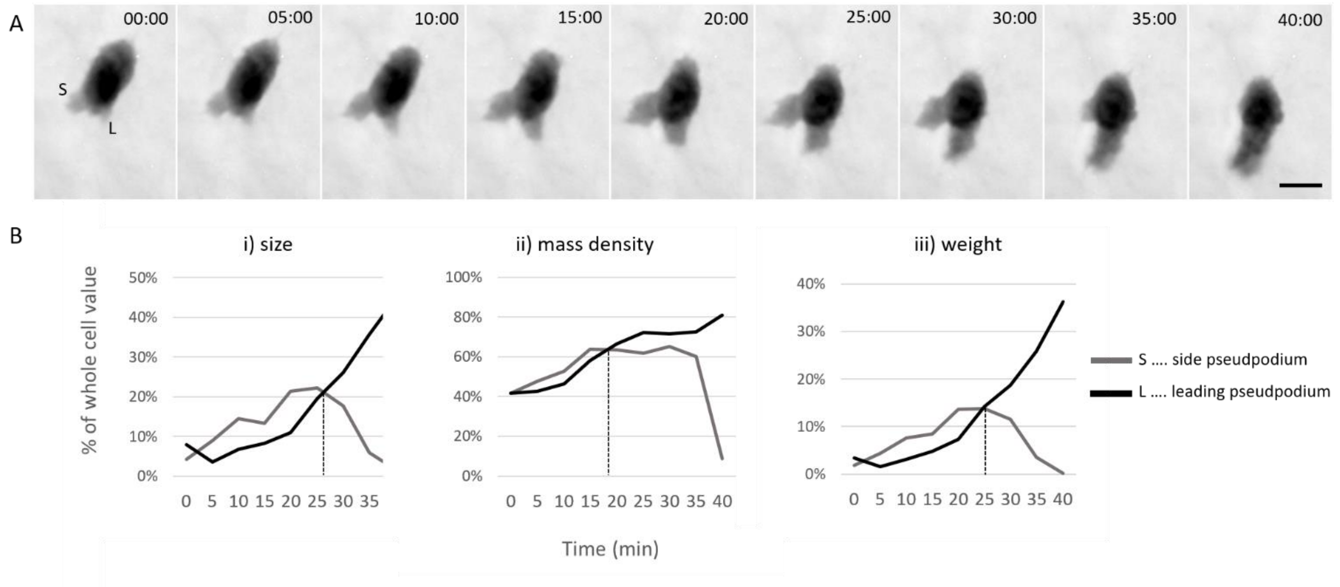

Figure S4. Analysis of mean cell mass in pseudopodia during amoeboid cell migration. Images acquired by CCHM of an amoeboid cell embedded in 3D collagen matrix were analysed to compute mean cell mass densities in whole cells and each pseudopodium. A: Analysed image sequence. B: The values for each pseudopodium are plotted as percentage of the values measured in the whole cell. The size (i) and weight (ii) differentiates the leading pseudopodium (L) from the side pseudopodium (S) at time 25 min, mean mass density (ii) already at time 20 min. Scale bar: 10 µm.

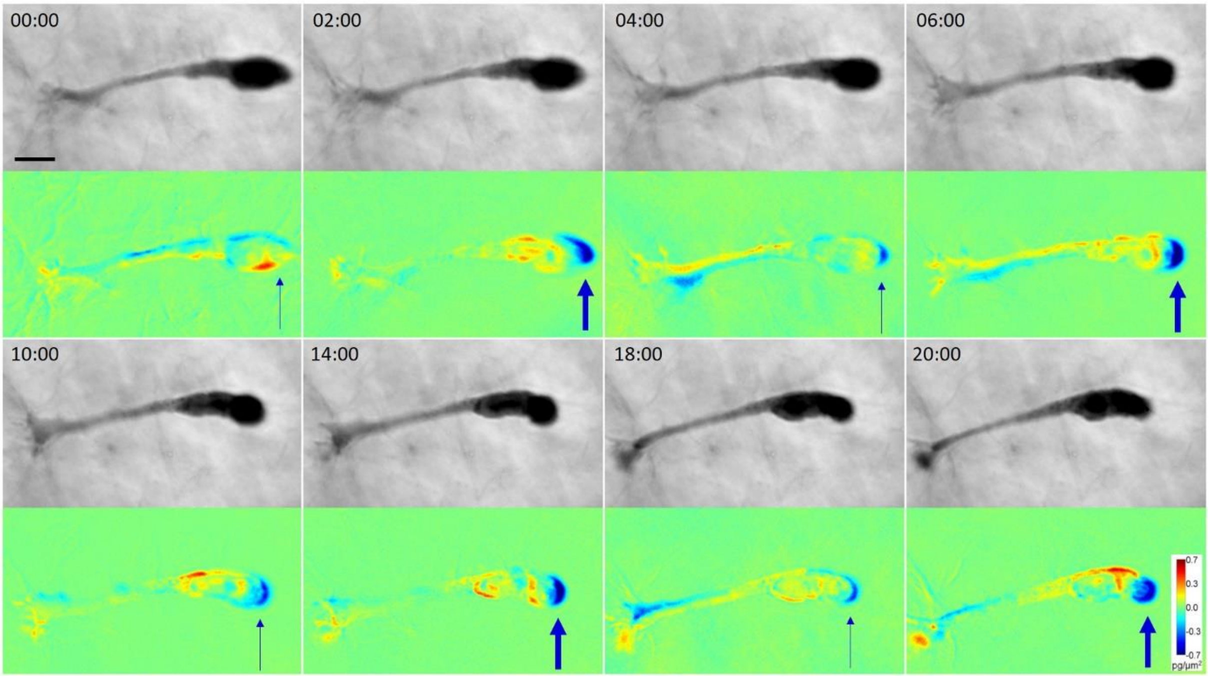

Figure S5: Cell migration dynamics demonstrated by DPD. Dynamic phase differences were calculated for an image sequence of a migrating mesenchymal cell. It can be noticed that the cell undergoes cycles of rear retraction, here clearly depicted by dark blue colour. The rear retraction succeeds a less dynamic phase with lower mass translocation, here shown as thin and thick arrows. Scale bar: 10 µm.

Supplementary videos

Supplementary video V1. A migration of mesenchymal cell within collagen matrix. *Cells were embedded within bovine collagen gel (1mg/ml) and observed using CCHM. Grey values were adjusted for best fibre contrast.*

Supplementary video V2. A migration of mesenchymal cell within collagen matrix. *Cells were embedded within bovine collagen gel (1mg/ml) and observed using CCHM. Grey values were adjusted for best fibre contrast. This video shows a similar situation as Suppl. Video V1.*

Supplementary video V3. A migration of mesenchymal cell within low density collagen matrix. *Cells were embedded in 0.5 mg/ml bovine collagen. The cell movement within low density collagen affects the overall architecture of collagen fibre matrix to higher degree then in higher density collagen (compare with video V1, V2). Grey values were adjusted for best fibre contrast.*

Supplementary video V4. Clustering of collagen fibres by a mesenchymal cell. *Cells were embedded within bovine collagen gel (1mg/ml) and observed using CCHM. Note that the vesicular material (black spots) is directed towards the very front pseudopodium, and a temporal appearance of blebs around nucleus where is the cell constricted (see also Supplementary video V6).*

Supplementary video V5. Clustering of collagen fibres by a mesenchymal cell. *This video is a detail from video V4. For details see text and Fig. 2.*

Supplementary video V6. Perinuclear blebbing during mesenchymal invasion. *This video is a detail from video V4. Membrane blebbing can be observed around the nucleus at sites of constriction.*

Supplementary video V7. Cell mass distribution during amoeboid invasion. *An amoeboid cell embedded in rat-tail collagen (1 mg/ml) was observed using CCHM. DPD were calculated during post-processing. The left and right part show the actual and subsequent image, respectively, DPD are shown in the middle.*

Supplementary video V8. Translocation of an amoeboid cell through a narrow pore. *An amoeboid cell embedded in rat-tail collagen (1 mg/ml) was observed using CCHM. This video demonstrates the dynamic cell body deformation during invasion through a narrow pore.*
